# HSP90β controls NLRP3 autoactivation

**DOI:** 10.1126/sciadv.adj6289

**Published:** 2024-02-28

**Authors:** Lotte Spel, Cyrielle Hou, Katerina Theodoropoulou, Léa Zaffalon, Zhuo Wang, Arinna Bertoni, Stefano Volpi, Michaël Hofer, Marco Gattorno, Fabio Martinon

**Affiliations:** ^1^ Department of Immunobiology, University of Lausanne, 155 Ch. des Boveresses, Epalinges 1066, Switzerland.; ^2^ Pediatric Unit of Immunology, Allergology, and Rheumatology, University Hospital of Lausanne, Lausanne, Switzerland.; ^3^ UOC Reumatologia e Malattie Autoinfiammatorie, IRCCS Istituto Giannina Gaslini, Genoa, Italy.; ^4^ DINOGMI, Università degli Studi di Genova, Genoa, Italy.

## Abstract

Gain-of-function mutations in NLRP3 are linked to cryopyrin-associated periodic syndromes (CAPS). Although NLRP3 autoinflammasome assembly triggers inflammatory cytokine release, its activation mechanisms are not fully understood. Our study used a functional genetic approach to identify regulators of NLRP3 inflammasome formation. We identified the HSP90β-SGT1 chaperone complex as crucial for autoinflammasome activation in CAPS. A deficiency in HSP90β, but not in HSP90α, impaired the formation of ASC specks without affecting the priming and expression of inflammasome components. Conversely, activating NLRP3 with stimuli such as nigericin or alum bypassed the need for SGT1 and HSP90β, suggesting the existence of alternative inflammasome assembly pathways. The role of HSP90β was further demonstrated in PBMCs derived from CAPS patients. In these samples, the pathological constitutive secretion of IL-1β could be suppressed using a pharmacological inhibitor of HSP90β. This finding underscores the potential of SGT1-HSP90β modulation as a therapeutic strategy in CAPS while preserving NLRP3’s physiological functions.

## Teaser

Suppressing HSP90β activity attenuates inflammasome activation in the presence of NLRP3 gain-of-function mutations.

## INTRODUCTION

Inflammation is an essential controlled process initiated by the innate immune system in response to pathogens, infection, and tissue damage. Among the sensors of pathogen and cellular insults, the cytoplasmic multiprotein complexes called inflammasomes have emerged as key players in innate immune responses (*1*, *2*). NLRP3 is probably the most studied inflammasome. Since its initial identification at the molecular level (*3*), inhibiting NLRP3 has shown promising results in over a hundred models of inflammatory diseases and conditions (*4*). These studies have highlighted the potential therapeutic value of targeting NLRP3 in human diseases. This is particularly relevant in the case of cryopyrin-associated periodic syndrome (CAPS), a rare form of inherited autoinflammatory disease caused by gain-of-function mutations in NLRP3 (*5*). These mutations trigger NLRP3 autoactivation, possibly by inducing activating conformational changes, or through other mechanisms, such as interference with inhibitory processes. CAPS disease is characterized by recurrent or continuous attacks of fever and systemic inflammation that affect various parts of the body, including the skin, joints, eyes, bones, muscles, and the central nervous system. These symptoms occur because of the increased secretion of interleukin-1β (IL-1β), mediated by aberrant inflammasome activity (*3*, *6*). The relevance of the pathway was demonstrated with the inhibition of IL-1 signaling that relieved most symptoms in CAPS (*7*).

NLRP3 belongs to the family of nucleotide-binding oligomerization domain and leucine-rich repeat–containing proteins (NLRs) (*8*). NLRs in vertebrates and plants (*9*) have been shown to rapidly detect pathogens or loss of cellular integrity, either directly or indirectly, and trigger the oligomerization of active NLR complexes, such as the inflammasomes. For example, exposure of macrophages to nigericin, alum, or crystals of monosodium urate induces activation of NLRP3 (*10**–**12*). Upon engagement, NLRP3 oligomerizes via its central NACHT domain and recruits the adaptor apoptosis-associated speck-like protein containing a caspase-recruitment domain (ASC) via its N-terminal pyrin domain (PYD). This results in the activation of the protease caspase-1, which cleaves the proinflammatory cytokines IL-1β and IL-18 into their active forms. Caspase-1 also cleaves other substrates that mediate pyroptosis, a type of cell death caused by the pore-forming cleavage product of gasdermin D (GSDMD) (*13*, *14*). Recent years have shed some light on the mechanism involved in inflammasome oligomerization (*15*). In particular, structural studies have shown how the NLRP3 inflammasome can form an inactive NLRP3 structure in the form of a cage that prevents its autoactivation (*16*, *17*). Moreover, components involved in the activation, such as the NIMA-related kinase 7 (NEK7), have been identified as NLRP3 binding partners that, at least in some context, are required for inflammasome activity (*18**–**21*).

Among other possible regulators of NLRP3, a two-hybrid interaction screen initially identified SGT1 (suppressor of the G_2_ allele of SKP1) as an interactor of NLRP3 LRR (*22*). Similarly, SGT1 has been proposed to regulate other NLRs in mammals such as NOD1 (*23*). Together with its partner HSP90, SGT1 is a known regulator of several cellular pathways, including aspects of yeast kinetochore assembly (*24*). However, most studies focused on demonstrating the role of this co-chaperone complex in plant NLR genes. Genetic studies in plants suggested that SGT1, together with HSP90, were involved in maintaining NLRs in a recognition-competent state while preventing aberrant activation of downstream pathways (*25**,* *26*). However, plants and vertebrate NLRs are not evolutionarily related (*27*), and the question of whether, as a consequence of convergent evolution, the SGT1-HSP90 complex also regulates mammalian NLRs and NLRP3, in particular, is still unclear. Initial studies used HSP90 inhibitors that showed robust inhibition of inflammasome engagement (*22*). However, these inhibitors strongly affect several kinases involved in inflammation (*28*), including pathways required for inflammasome priming (*29*), challenging, therefore, the conclusion that HSP90 and SGT1 could be directly involved in inflammasome assembly.

The mechanisms underlying the activation of NLRP3 inflammasome are not well understood. It would be beneficial to comprehend how different stimuli engage NLRP3 in distinct ways to develop effective therapeutic strategies. In this study, we aimed to identify regulators of NLRP3 inflammasome activation by investigating the mechanisms driving NLRP3 activation in CAPS. We conducted an unbiased genome-wide knockout screen using a cellular model recapitulating the inflammatory cell death mediated by gain-of-function NLRP3. Through this screen, we identified two crucial proteins, SGT1 and HSP90β, that are necessary for the formation of the NLRP3 autoinflammasome. In vertebrates, there are two cytoplasmic paralog of HSP90, HSP90α and HSP90β, which exhibit differential expression in embryonic and adult tissues, as well as under stressful conditions. Although they share similar functional activities within chaperone complexes, it has been suggested that HSP90β may have a few specific client proteins (*30*). In line with this possibility, the deficiency of HSP90α did not affect NLRP3 assembly, enabling us to investigate the role of HSP90β independently from fully impaired HSP90 function. Our observations revealed that the absence of SGT1 and HSP90β impaired NLRP3 inflammasome activity in most CAPS models with specific mutations. However, the effect was only modest when NLRP3 was activated by external stimuli like alum or nigericin. In addition, pharmacological inhibitors targeting HSP90β prevented the abnormal secretion of IL-1β from peripheral blood mononuclear cells (PBMCs) isolated from CAPS patients, except in cases of the most severe form of the disease.

In conclusion, our findings demonstrate that SGT1 and HSP90β are integral components of the NLRP3 inflammasome. Furthermore, our study highlights a subgroup of patients who could benefit from therapies targeting HSP90β.

## RESULTS

### HSP90β and SGT1 are required for NLRP3 activation in CAPS

**Fig. 1.**
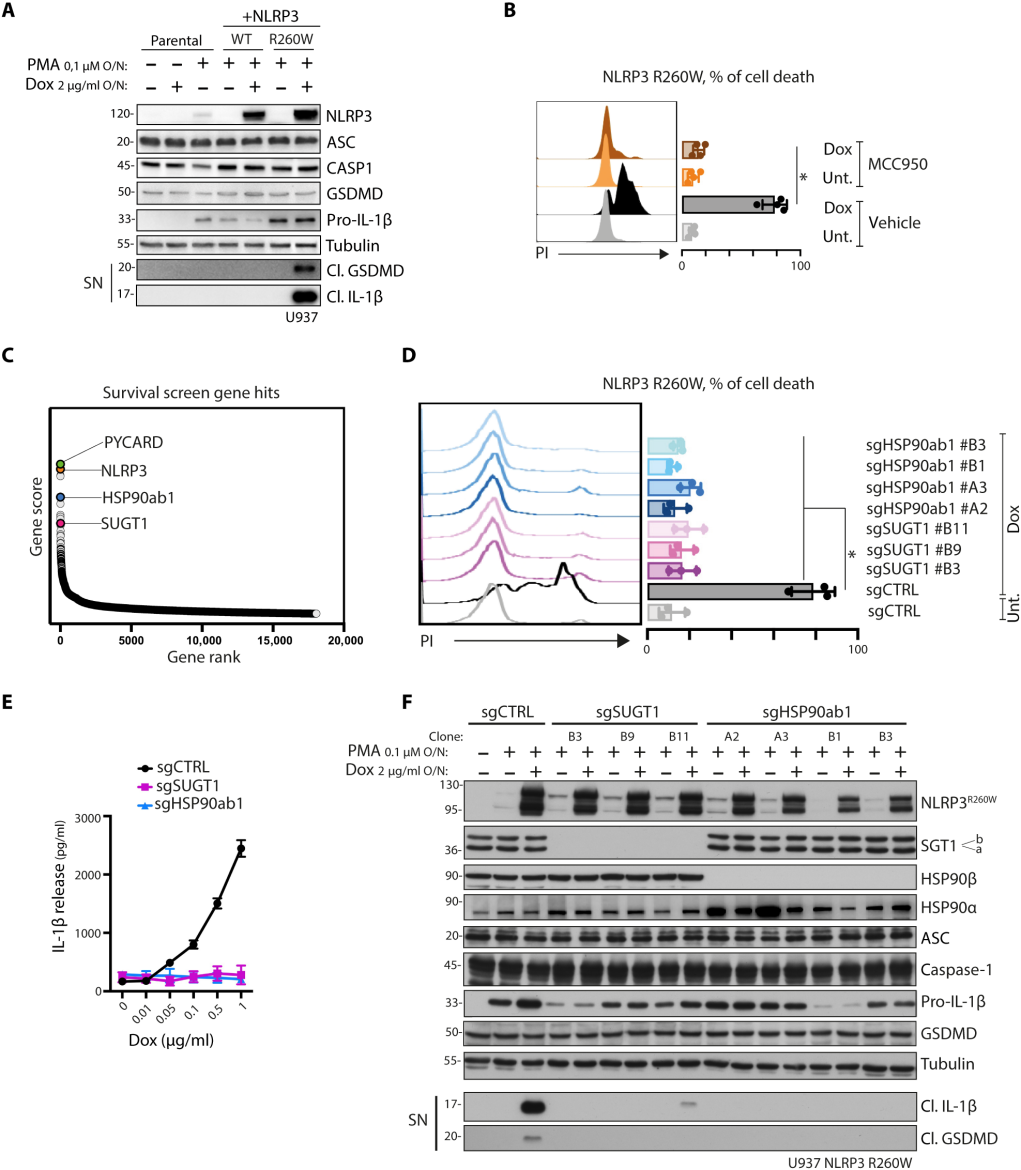
HSP90β and SGT1 are required for NLRP3 activation in CAPS. (**A**) Inflammasome activation in U937 cells expressing doxycycline (Dox)–inducible NLRP3 WT or NLRP3 R260W. Cells are treated with phorbol myristate acetate (PMA) and doxycycline overnight (O/N). Protein expression and release of inflammasome activation markers, cleaved GSDMD and cleaved IL-1β, in the supernatant (SN) are measured by Western blot. Pro, precursor; Cl., cleaved. Tubulin is used as a loading control. (**B**) Cell death analysis of U937 cells expressing doxycycline-inducible NLRP3 R260W. Cells are treated with doxycycline for 24 hours in the absence or presence of MCC950, and propidium iodide (PI)–positive cells are measured by flow cytometry. (**C**) Results of genome-wide knockout screen in U937 NLRP3 R260W. Selected gene hits are indicated in the graph. (**D**) Cell death analysis of SUGT1- and HSP90ab1-deficient U937 NLRP3 R260W clones. Cells are treated with doxycycline for 24 hours, and PI-positive cells are measured by flow cytometry. (**E**) Inflammasome activation in U937 NLRP3 R260W control cells (sgCTRL) or lacking SGT1 (sgSUGT1) or HSP90β (sgHSP90ab1). Cells are treated with PMA and doxycycline overnight. Release of IL-1β in the supernatant is quantified by ELISA. (**F**) Inflammasome activation in U937 NLRP3 R260W control cells (sgCTRL) or clones lacking either SGT1 (sgSUGT1) or HSP90β (sgHSP90ab1). Cells are treated with PMA and doxycycline overnight. Protein expression and release of inflammasome activation markers are measured by Western blot. Western blots are representative of three independent experiments. Fluorescence-activated cell sorting data are obtained from minimum three independent experiments, represented as means ± SD, and tested for statistical significance using nonparametric Mann-Whitney *U* or nonparametric Kruskal-Wallis test (*P* ≤ 0.05 is considered significant and indicated with *).

The most common mutation in CAPS disease is R260W, located within the NACHT domain. In this study, we used a cellular model of CAPS using U937 cell lines with doxycycline-inducible expression of NLRP3 wild type (WT) or CAPS (R260W). Our findings demonstrate that expression of the R260W mutation, but not the WT, recapitulates inflammasome activation, as evidenced by the secretion of cleaved IL-1β and cleaved GSDMD (Fig. 1A). Moreover, we observed pyroptotic cell death induction in this model, which could be blocked by treatment with the NLRP3 inhibitor MCC950, confirming the direct involvement of the inflammasome (Fig. 1B). To identify the genes essential for inflammasome-induced cell death, we performed a survival screen using a genome-wide CRISPR library in the CAPS cellular model (fig. S1). The most enriched target genes within the surviving cell population were NLRP3 and PYCARD, encoding the inflammasome components NLRP3 and ASC, respectively (Fig. 1C and data S1 and S2). In addition, *HSP90ab1* and *SUGT1* emerged as statistically significant gene hits (Fig. 1C), encoding for HSP90β and SGT1, respectively. To validate the screen results, we generated SGT1- and HSP90β-deficient cells using newly designed single-guide RNA (sgRNA) sequences and confirmed that deficiencies in both HSP90β and SGT1 protected cells from inflammasome-mediated cell death (Fig. 1D).

Subsequently, we evaluated IL-1β production in HSP90β- and SGT1-deficient cells stimulated with increasing concentrations of doxycycline. While control cells exhibited increased IL-1β secretion, cells lacking HSP90β or SGT1 failed to secrete IL-1β (Fig. 1E), indicating impaired inflammasome activity. Furthermore, we confirmed that the expression of inflammasome components, including NLRP3, ASC, and caspase-1, as well as their substrates, pro–IL-1β, and GSDMD, remained unaffected by deficiencies in SGT1 or HSP90β (Fig. 1F). However, the release of cleaved IL-1β and cleaved GSDMD into the cell supernatant was significantly diminished upon deletion of HSP90β or SGT1 (Fig. 1F). The role of this complex in NLRP3 regulation is further supported by immunoprecipitation studies. These studies demonstrated the binding of SGT1 and HSP90β to both the NLRP3 WT and the NLRP3 CAPS R260W variant (fig. S2A). These observations suggest a functional and direct role of HSP90β or SGT1 in the proteolytic activation of the inflammasome complex.

### NLRP3 function is HSP90β and SGT1 selective

**Fig. 2.**
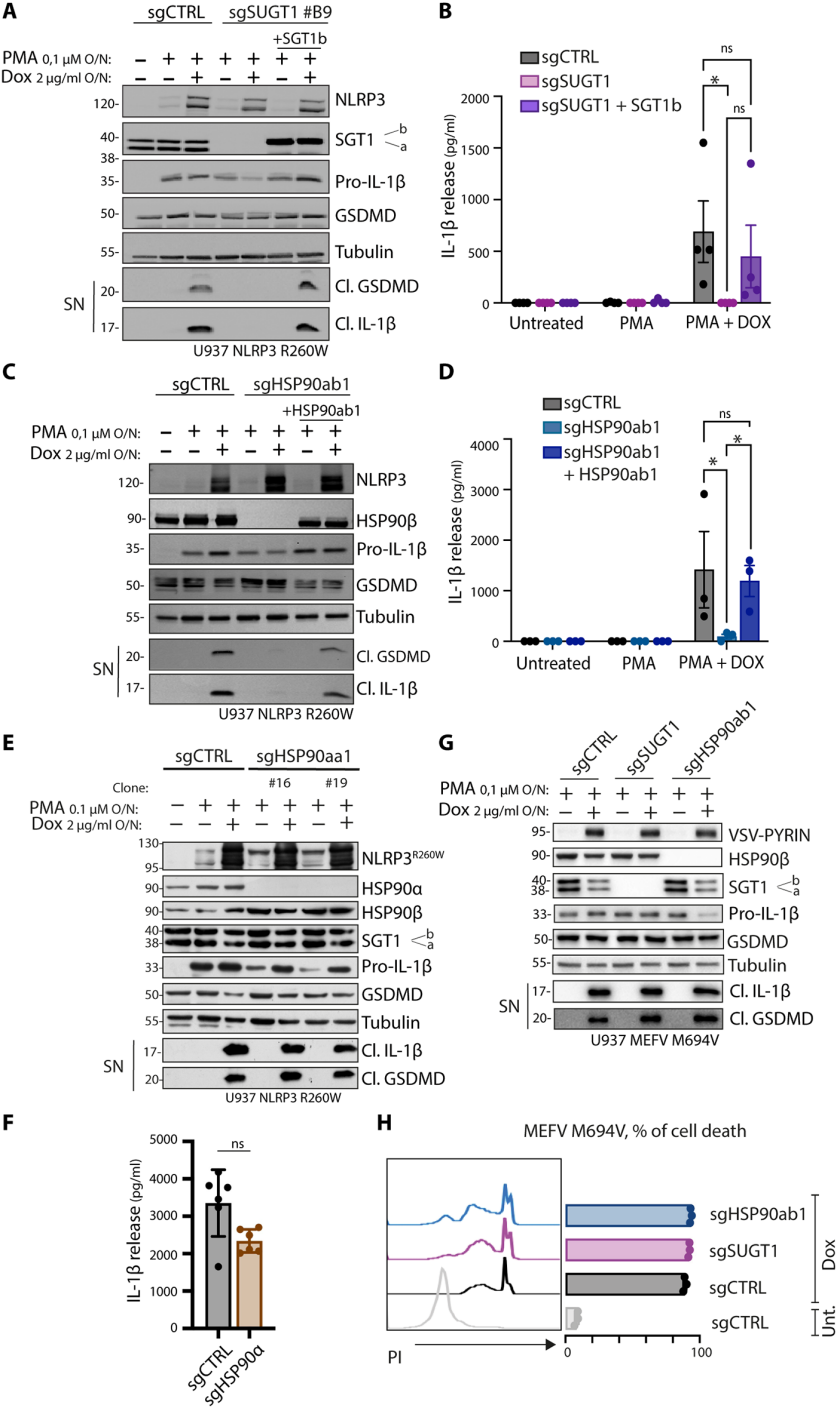
NLRP3 function is HSP90β and SGT1 selective. (**A** and **Β**) Inflammasome activation in U937 NLRP3 R260W control cells or depleted for SGT1 (sgSUGT1) or reconstituted with SGT1b. Cells are treated with PMA and doxycycline overnight. (A) Protein expression and release of inflammasome activation markers are measured by Western blot. (B) Release of cleaved IL-1β in the supernatant is quantified by ELISA. (**C** and **D**) Inflammasome activation in U937 NLRP3 R260W control cells or depleted for HSP90β (sgHSP90ab1) or reconstituted with HSP90ab1. Cells are treated with PMA and doxycycline overnight. (C) Protein expression and release of inflammasome activation markers are measured by Western blot. (D) Release of cleaved IL-1β in the supernatant is quantified by ELISA. (**E** and **F**) Inflammasome activation in U937 NLRP3 R260W control cells (sgCTRL) or lacking HSP90α (sgHSP90aa1). Cells are treated with PMA and doxycycline overnight. (E) Protein expression and release of inflammasome activation markers are measured by Western blot. (F) Release of cleaved IL-1β in the supernatant of doxycycline-treated cells is quantified by ELISA. (**G**) Inflammasome activation in U937 MEFV M694V control cells or depleted for SGT1 (sgSUGT1) or HSP90β (sgHSP90ab1). Cells are treated with PMA and doxycycline overnight. Protein expression and release of inflammasome activation markers are measured by Western blot. (**H**) Cell death analysis of U937 MEFV M694V control cells or depleted for SGT1 (sgSUGT1) or HSP90β (sgHSP90ab1). Cells are treated with doxycycline for 24 hours, and PI-positive cells are measured by flow cytometry. Western blots are representative of three or four independent experiments. ELISA data are obtained from three or four independent experiments, represented as means ± SD, and tested for statistical significance using the nonparametric Mann-Whitney *U* test (*P* ≤ 0.05 is considered significant and indicated with *; ns, not significant).

In monocytes, SGT1 expresses two isoforms, namely, SGT1a and SGT1b (*31*), which are both depleted in deficient populations (Fig. 1F). To investigate the involvement of these isoforms in NLRP3 activation, we reconstituted SGT1-deficient cells with stable constructs expressing each isoform. Our findings revealed that both SGT1a (fig. S2B) and SGT1b (Fig. 2, A and B) restored the cleavage of GSDMD and IL-1β secretion, indicating their redundant contribution to inflammasome activity. Similarly, in HSP90β-deficient cells, reconstitution with a construct expressing HSP90β restored inflammasome activity (Fig. 2, C and D). HSP90α and HSP90β are closely related paralogs that may have similar molecular functions. In the context of HSP90β deficiency, we observed increased expression of HSP90α (Fig. 1F). Consequently, we examined whether HSP90α could contribute to NLRP3 activation. However, our results demonstrated that HSP90α deficiency did not affect inflammasome activity, as evidenced by the cleavage of GSDMD and the release of IL-1β observed through Western blot analysis (Fig. 2E) and enzyme-linked immunosorbent assay (ELISA) (Fig. 2F). These findings suggest that HSP90α and HSP90β differ in their ability to regulate inflammasome function.

Various inflammasomes share common components such as ASC, caspase-1, and substrates like IL-1β. To assess the role of SGT1 and HSP90β in these shared pathways, we investigated their involvement in the Pyrin inflammasome. Pyrin is encoded by the *MEFV* gene and is associated with gain-of-function mutations in patients with familial Mediterranean fever (FMF). Using a model of FMF caused by the MEFV M694V mutation (*32*), we found that neither HSP90β nor SGT1 contributed to the secretion of IL-1β (Fig. 2G) or induction of pyroptotic cell death (Fig. 2H). These observations suggest that HSP90β and SGT1 may specifically affect upstream events related to NLRP3 engagement.

### SGT1 and HSP90β facilitate inflammasome speck formation in NLRP3 CAPS model

**Fig. 3.**
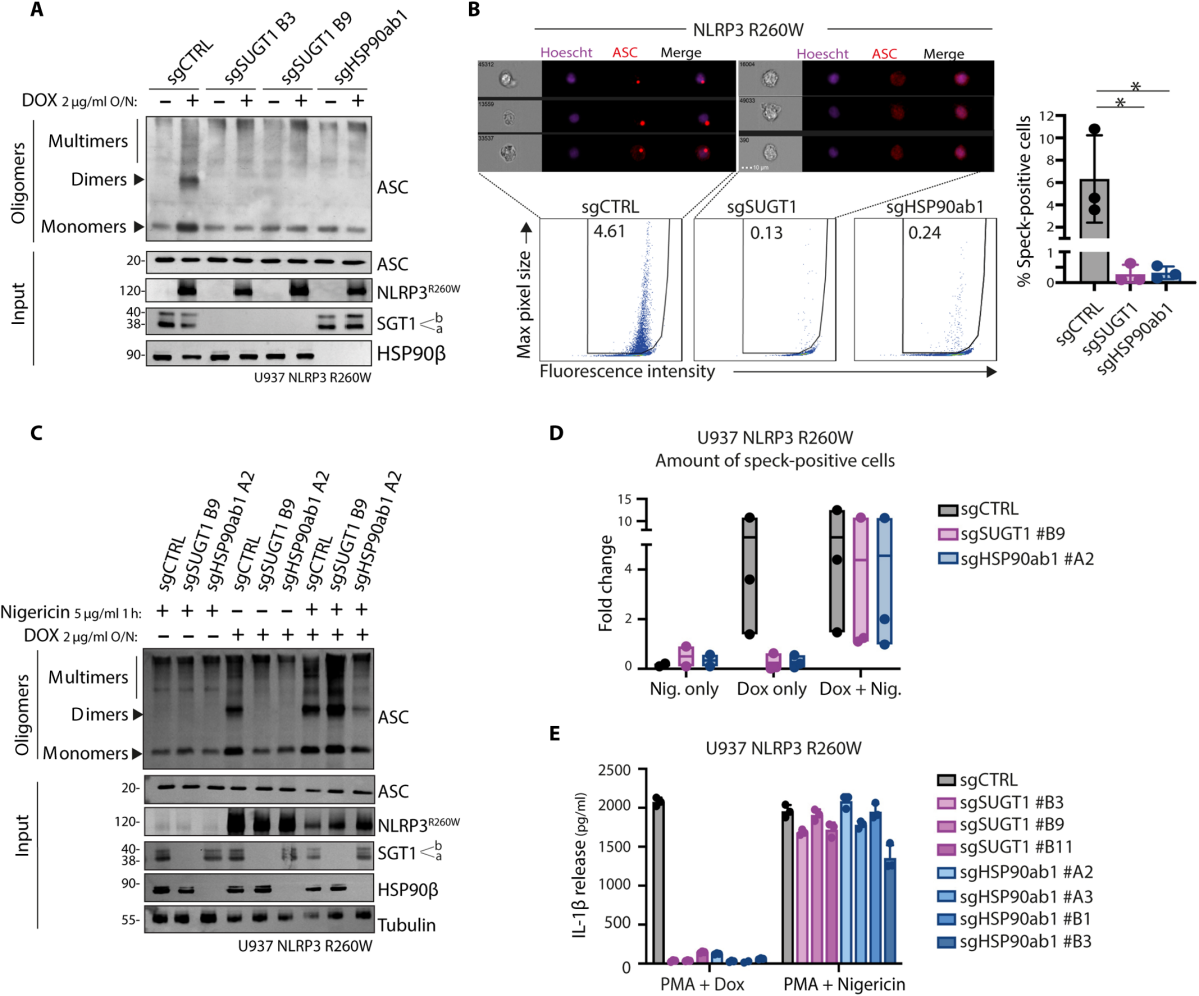
SGT1 and HSP90β facilitate inflammasome speck formation in NLRP3 CAPS model. (**A**) ASC oligomerization in U937 NLRP3 R260W control cells (sgCTRL) or lacking SGT1 (sgSUGT1) or HSP90β (sgHSP90ab1). Cells are treated with doxycycline overnight or left untreated. Protein lysates are cross-linked and analyzed by Western blot. (**B**) SGT1 (sgSUGT1)– or HSP90β (sgHSP90ab1)–deficient U937 NLRP3 R260W clones or control populations (sgCTRL) were treated with doxycycline and z-vad-fmk for 24 hours and intracellularly stained using anti-ASC antibodies. Hoechst was added to stain nuclei. Cells are measured using imaging flow cytometry. Representative images with ASC specks in sgCTRL or ASC diffuse pattern in sgSUGT1 cells are shown. Bottom shows the quantification of ASC speck formation in the tested conditions. (**C** and **D**) ASC oligomerization in U937 NLRP3 R260W control cells (sgCTRL) or lacking SGT1 (sgSUGT1) or HSP90β (sgHSP90ab1). Cells are treated with nigericin (Nig.), doxycycline, or both. Cells were either lysed for protein cross-linking and analyzed by Western blot (C) or fluorescently stained and analyzed by imaging flow cytometry for ASC speck formation (D). (**E**) Inflammasome activation in U937 NLRP3 R260W control cells (sgCTRL) or lacking SGT1 (sgSUGT1) or HSP90β (sgHSP90ab1). Cells are treated either with PMA and doxycycline or with PMA and nigericin. The release of cleaved IL-1β in the supernatant is quantified by ELISA. Data are obtained from three independent experiments, and represented as means ± SD, and tested for statistical significance using the nonparametric Mann-Whitney *U* test (*P* ≤ 0.05 is considered significant and indicated with *).

Without priming or signal 1, NLRP3 R260W can initiate ASC oligomerization, a hallmark of inflammasome assembly. Through cross-linking analysis of ASC oligomeric structures, we observed that the formation of these oligomers required HSP90β and SGT1 (Fig. 3A). In addition, ASC oligomers can be visualized as ASC specks using imaging flow cytometry with anti-ASC antibodies. Quantifying these specks upon NLRP3 R260W expression revealed significant impairment in speck formation in HSP90β- and SGT1-deficient cells compared to control cells (Fig. 3, B and C). These results suggest that cells depleted of SGT1 or HSP90β cannot elicit the oligomerization of NLRP3 R260W with ASC into a functional inflammasome structure.

Subsequently, we tested whether NLRP3 R260W would remain responsive to exogenous activation stimuli, such as nigericin, under these conditions. We observed that in the absence of HSP90β or SGT1, nigericin stimulation of NLRP3 R260W restored ASC oligomerization (Fig. 3C) and speck formation (Fig. 3D). These observations suggest that while the absence of SGT1 and HSP90β impairs autoactivation, it does not diminish NLRP3 functionality, as measured by the ability of NLRP3 R260W to respond to external stimuli. Pulse-chase experiments showed no significant differences in the stability of NLRP3 R260W in the absence of SGT1 and HSP90β, further indicating that SGT1 and HSP90β may not directly function by impairing the overall folding of NLRP3 (fig. S3A). Together, these data suggest that the activation of NLRP3 by the gain-of-function mutation or nigericin stimulation may rely on different molecular mechanisms.

To further investigate this phenomenon, we tested whether the HSP90β- and SGT1-deficient cell populations could activate inflammasome in the absence of CAPS NLRP3 expression. By using PMA (phorbol myristate acetate) to induce priming, we triggered the expression of endogenous NLRP3 and the pro–IL-1β substrate. Under these conditions, nigericin stimulation of endogenous NLRP3 could induce the secretion of mature IL-1β independently of SGT1 and HSP90β (Fig. 3E).

### Stimuli-mediated NLRP3 activation does not rely on the HSP90b-SGT1 pathway

**Fig. 4.**
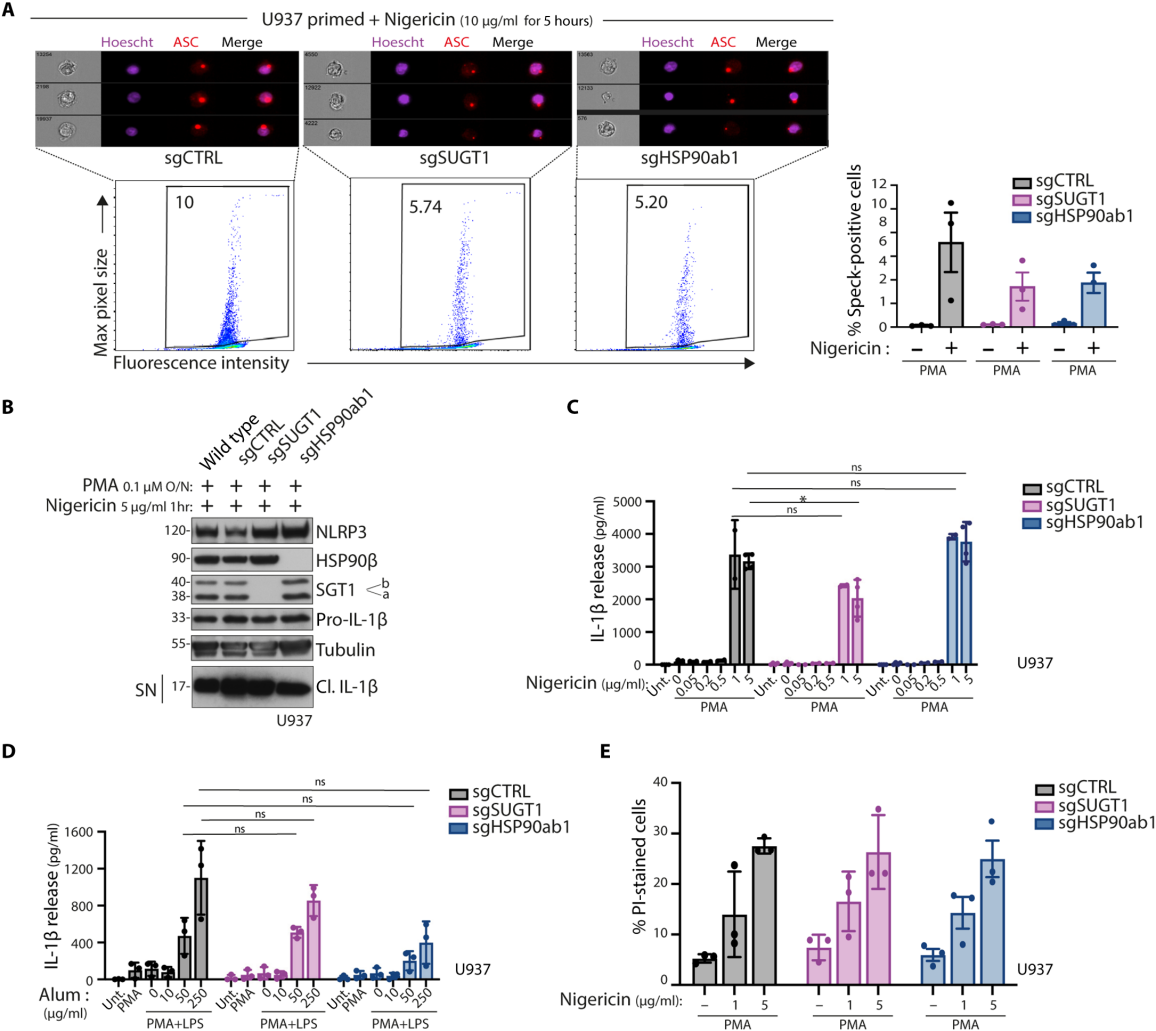
Stimuli-mediated NLRP3 activation does not rely on the HSP90β-SGT1 pathway. (**A** and **B**) Inflammasome activation in U937 control cells (sgCTRL) or lacking SGT1 (sgSUGT1) or HSP90β (sgHSP90ab1). Cells are treated either with PMA, z-vad-fmk overnight, or nigericin as indicated for 5 hours and intracellularly stained using anti-ASC antibodies and Hoechst to stain the nucleus. Representative images with ASC specks in cells are shown. (A) Cells are analyzed using imaging flow cytometry. (B) Protein expression and release of inflammasome activation markers are measured by Western blot. (**C**) Inflammasome activation in U937 control cells (sgCTRL) or lacking SGT1 (sgSUGT1) or HSP90β (sgHSP90ab1). Cells are treated with PMA overnight and subsequently stimulated with nigericin for 1 hour. (C) and (**D**) Inflammasome activation in U937 control cells (sgCTRL) or lacking SGT1 (sgSUGT1) or HSP90β (sgHSP90ab1). Cells are treated with PMA or PMA and LPS, as indicated, overnight and subsequently stimulated with indicated concentrations of nigericin for 1 hour (C) or alum for 6 hours (D). Release of cleaved IL-1β in the supernatant is quantified by ELISA. (**E**) Inflammasome activation in U937 control cells (sgCTRL) or lacking SGT1 (sgSUGT1) or HSP90β (sgHSP90ab1). Cells are treated with PMA overnight and subsequently stimulated with indicated concentrations of nigericin for 1 hour. Cell death was analyzed by PI-positive staining and measured by flow cytometry. Data are obtained from three independent experiments, represented as means ± SD, and tested for statistical significance using nonparametric Mann-Whitney *U* test (*P* ≤ 0.05 is considered significant and indicated with *).

To further examine the role of HSP90β and SGT1 in response to exogenous stimuli, we investigated their contribution to inflammasome assembly in parental U937 cells without an inducible construct. We used two activators of the NLRP3 inflammasome: the strong NLRP3 inducer nigericin and the mild NLRP3 inducer aluminum hydroxide (alum). After overnight priming with PMA, we found that nigericin was still able to elicit the formation of ASC specks in SGT1- or HSP90β-deficient cells compared to controls (Fig. 4A). We monitored IL-1β and found that cells deficient in SGT1 or HSP90β were still capable of secreting IL-1β (Fig. 4B). Quantifying protein secretion by ELISA showed a slight decrease in SGT1-deficient cells only after the highest concentration of nigericin (Fig. 4C). Similarly, IL-1β secretion stimulated by alum treatment was not statistically affected by the depletion of SGT1 or HSP90β (Fig. 4D). Moreover, cell death triggered by nigericin was not affected by SGT1 or HSP90β deficiency (Fig. 4E). These results suggest that while SGT1 and HSP90β are required in the CAPS model with active NLRP3, they are not essential components for integrating exogenous stimuli. These findings indicate that disrupting SGT1 or HSP90β function may offer a possibility to specifically target NLRP3 disease activity without affecting the WT NLRP3 response.

### Pharmacological inhibition of HSP90β reduces IL-1β secretion in CAPS

**Fig. 5.**
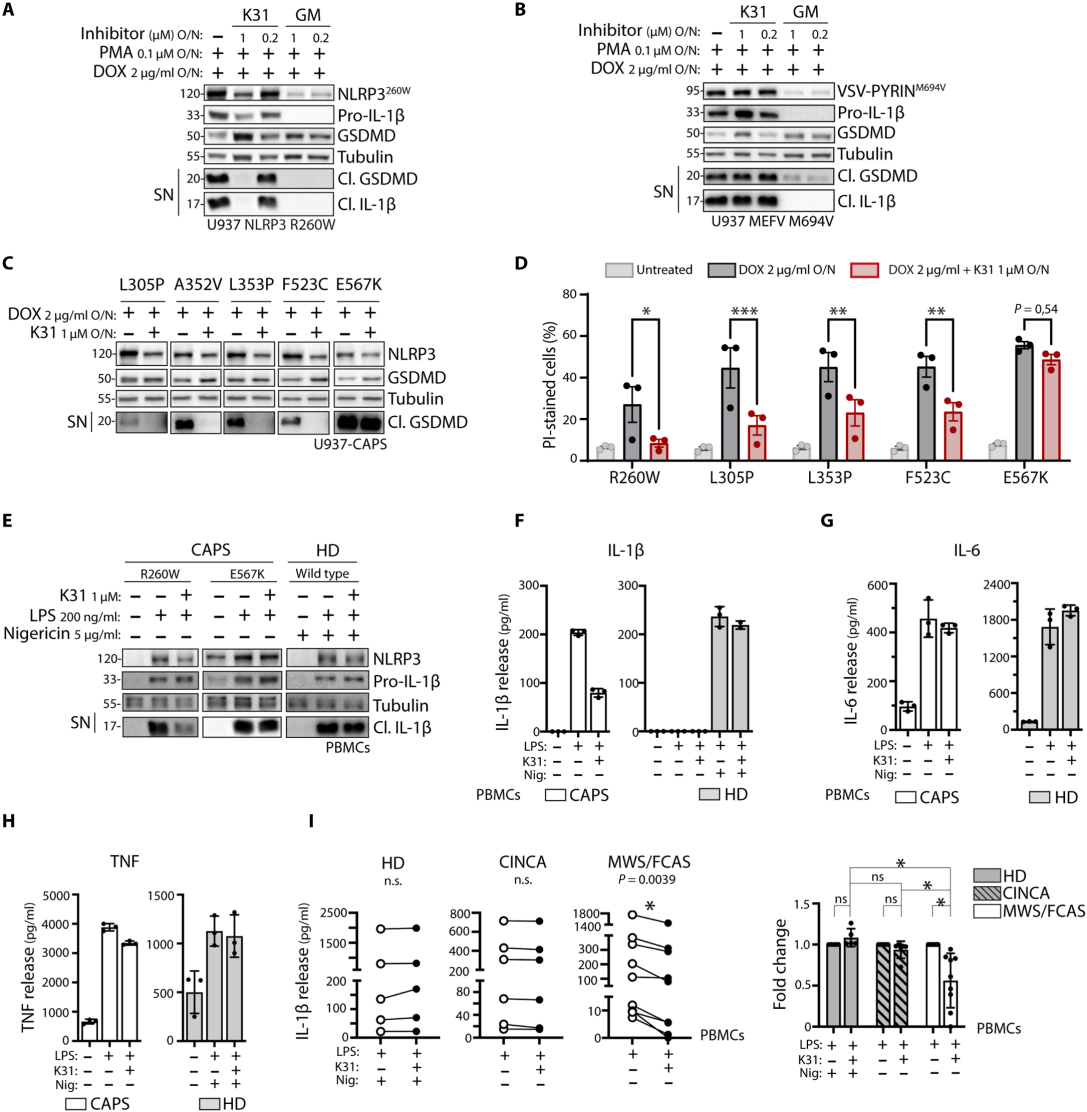
Pharmacological inhibition of HSP90β reduces IL-1β secretion in CAPS. (**A** and **B**) Cells are treated with PMA and doxycycline overnight in the absence or presence of KUNB31 (K31) or GM. Protein expression and release of inflammasome activation markers are measured by Western blot. Inflammasome activation in U937 cells expressing doxycycline-inducible NLRP3 R260W (A) or MEFV M694V (B). (**C** and **D**) Inflammasome activation in U937 cells expressing doxycycline-inducible NLRP3 mutants. Cells are treated with doxycycline overnight. Protein expression and release of inflammasome activation markers are measured by Western blot (C). Cell death in representative CAPS models was analyzed by PI-positive staining and measured by flow cytometry (D). (**E** to **H**) Inflammasome activation in healthy donor (HD)– or patient-derived PBMCs. Cells are treated with KUNB31 for 30 min or left untreated, after which LPS is added for 2 hours. For healthy donor PBMCs, nigericin is added for the last 45 min. Protein expression and release of inflammasome activation markers are measured by Western blot (E). Release of cleaved IL-1β (F), IL-6 (G), and TNF (H) in the supernatant is quantified by ELISA. (**I**) Inflammasome activation in PBMCs derived from healthy donors, CINCA patients, or MWS/FCAS patients. Cells are treated with KUNB31 for 30 min or left untreated, after which LPS is added for 2 hours. For healthy donor PBMCs, nigericin is added for the last 45 min. Release of cleaved IL-1β in the supernatant is quantified by ELISA, shown as raw data or fold decrease. ELISA data are obtained from three independent experiments, represented as means ± SD, and tested for statistical significance using paired *t* test or two-way analysis of variance (ANOVA) (*P* ≤ 0.05 is considered significant and indicated with *).

Common HSP90 inhibitors, such as geldanamycin (GM), do not differentiate between HSP90α and HSP90β, targeting both cytosolic isoforms simultaneously. However, recent advancements have led to the development of a highly specific HSP90β inhibitor called KUNB31 (*33*). Treatment with 1 μΜ KUNB31 in the CAPS model resulted in impaired R260W NLRP3-induced inflammasome activity (Fig. 5A). Unlike GM, which affected the expression of various components, KUNB31 had minimal impact on the expression of inflammasome components and substrates (Fig. 5A). Moreover, KUNB31, while impairing NLRP3 R260W, did not impair its ability to respond to nigericin (fig. S3B). The specificity of HSP90β inhibition by KUNB31 was further demonstrated in the FMF-Pyrin model, which remained unaffected by KUNB31, unlike in the presence of the broad nonspecific activity of GM (Fig. 5B).

To enhance our understanding, we explored various mutations, primarily in the NLRP3 NACHT domain, which are known to cause CAPS. We developed U937 models for some of the most common mutations, following the methodology previously described for R260W. These mutations demonstrated consistent inflammasome activity, evidenced by GSDMD maturation and the induction of cell death. Treatment with KUNB31 significantly reduced GSDMD cleavage in all modeled mutations, except for E567K (Fig. 5C). Similarly, KUNB31 decreased cell death induced by the expression of CAPS variants, with the exception of those harboring the E567K mutation (Fig. 5D). The spectrum of CAPS diseases encompasses clinical entities that display varying severity. Familial cold auto-inflammatory syndrome (FCAS) and Muckle-Wells syndrome (MWS) present with milder symptoms, while neonatal-onset multisystem inflammatory disorder (NOMID), also known as chronic infantile neurologic cutaneous and articular syndrome (CINCA), displays a more severe phenotype with neurological involvement and bone dysplasia (*6*). To confirm the relevance of our cellular models, we tested inflammasome activation in PBMCs from healthy controls and CAPS patients, including an MWS patient carrying the R260W mutation and a CINCA patient with the E567K mutation. The cells were primed with lipopolysaccharide (LPS) treatment to induce transcriptional expression of NLRP3 and pro–IL-1β (*3*). This led directly to autoinflammasome activation in CAPS cells, as observed by the release of IL-1β (Fig. 5E). Healthy donor PBMCs required additional stimulation with nigericin to activate the inflammasome. PBMCs carrying the R260W mutation were sensitive to HSP90β inhibition, resulting in decreased IL-1β release upon treatment with KUNB31. In contrast, HSP90β inhibition in PBMCs carrying the E567K mutation or in healthy donor PBMCs treated with nigericin did not significantly reduce IL-1β secretion, supporting our findings using the engineered CAPS cells.

Furthermore, we quantified cytokine secretion by ELISA in the supernatant of PBMCs from a CAPS patient harboring the R260W mutation and healthy donors. We confirmed that KUNB31 specifically impaired autoinflammasome-mediated IL-1β release in R260W PBMCs (Fig. 5F), while the release of LPS-mediated IL-6 (Fig. 5G) and tumor necrosis factor (TNF) (Fig. 5H) remained unaffected.

Similarly, we tested HSP90β inhibition in PBMCs derived from various CAPS patients with different mutations (table S1). Our study included five healthy donors, six patients with severe disease (CINCA), and nine patients with mild/intermediate disease (six MWS and three FCAS patients). HSP90β inhibition had no impact on healthy donor PBMCs treated with nigericin. However, treatment with KUNB31 reduced constitutive IL-1β release in MWS- or FCAS-derived PBMCs, but not in CINCA-derived PBMCs (Fig. 5I). These results confirmed the selectivity of HSP90β in the context of CAPS-associated NLRP3 in a physiological setting and suggest a distinct separation in the regulation of gain-of-function NLRP3 autoinflammasome based on disease severity.

## DISCUSSION

In this study, we have investigated the molecular mechanism of inflammasome activation by CAPS-associated NLRP3 variants. Through an unbiased functional screen, we found that HSP90β and SGT1 are crucial for human NLRP3 autoinflammasome complex formation in the context of CAPS disease. The interaction between NLRP3 and SGT1 was previously identified in a two-hybrid screen using NLRP3 LRR as bait (*3*). Furthermore, we recently established a correlation between the functionality of NLRP3 splice variants within the LRR and their ability to interact with SGT1 (*34*). However, these previous studies did not determine the specific inflammasome-activating conditions that depend on this co-chaperone complex. Here, we demonstrate that in the context of potent activation, like in the presence of nigericin, inflammasome activity was independent of SGT1 presence. Likewise, we observed that particulate elements like alum exhibited a slightly diminished ability to induce inflammasome activation in the absence of SGT1 or HSP90β. This suggests that the presence of the SGT1-HSP90β complex may not be indispensable for NLRP3 engagement under these specific conditions. However, it is still possible that the complex is necessary for achieving optimal activation under certain circumstances. In support of this, our genetic deletion and reconstitution experiments clearly show that most CAPS-associated NLRP3 variants rely on the presence of both SGT1 and HSP90β for the spontaneous assembly of the inflammasome. This finding aligns with plant genetic studies, which have demonstrated that the SGT1-HSP90 complex plays a crucial role in maintaining plant NLR proteins in a state that enables recognition, a mechanism necessary for activating multiple NLRs across various plant species (*26*, *35*). The conservation of these mechanisms between plants and mammals, despite the absence of NLRs in other animals such as *Caenorhabditis elegans* or insects, suggests that the roles of SGT1 and HSP90 in innate immunity were independently established in different evolutionary lineages. This convergent evolution may reflect inherent biochemical constraints underlining LRR and the oligomerization of NACHT-like domains.

When endogenous NLRP3 priming and expression were absent, treatment with nigericin circumvented the need for SGT1 and HSP90β, resulting in the complete restoration of NLRP3 activation in the CAPS variant (Fig. 3, C and D). This observation suggests that different engagement mechanisms are initiated depending on the specific stimuli involved, and defects in SGT1 or HSP90β do not hinder the competence for activation but specifically regulate spontaneous autoinflammasome formation. The presence of alternative activation mechanisms is in line with the observation that while most CAPS mutations relied on SGT1 and HSP90β, a few, such as E567K, did not. This suggests that these mutations might trigger a different self-activation mechanism. One possible explanation is that stronger signals or mutations that cause more severe symptoms are less dependent on SGT1 and HSP90β. In the context of abnormal autoactivation of NLRP3, SGT1 and HSP90β are required, but when robust oligomerization is involved, it could bypass the need for SGT1 and HSP90β by using an alternative pathway.

We found that NLRP3 specifically depends on HSP90β, while HSP90α has no impact on inflammasome assembly. It is commonly assumed that these paralogs have identical functions. However, studies have indicated that although they essentially share overlapping and redundant client specificities, isoform-specific functions have been described (*36*, *37*). In Arabidopsis, various members of the HSP90 family have been shown to have distinct roles in different NLR-mediated immune responses. In a genetic model containing an NLR variant called SNC1, HSP90.3 was identified as a regulator of SNC1 (*38*). Conversely, mutations in HSP90.2 specifically impaired pathogen recognition by the NLR protein RPM1 (*39*). These divergent phenotypes observed in HSP90.2 and HSP90.3 mutant alleles indicate that plant HSP90 paralogs may exhibit variations in NLR client specificities or preferences, similar to what we observed in human NLRP3.

The specificity of HSP90β for NLRP3 presents a promising opportunity for targeted therapeutic interventions. Previous efforts using broad HSP90 inhibitors like GM have affected multiple pathways regulated by these chaperones, including tyrosine kinases and nuclear receptors (*40*). However, the use of isoform-selective inhibitors that can selectively target specific pathways, such as NLRP3 in CAPS, could avoid the drawbacks and toxicity associated with current HSP90 inhibitors, offering an exciting option in CAPS treatment. In addition, our data suggest that this strategy may reduce autoinflammation while still allowing NLRP3 engagement and activation in response to specific stimuli. This approach could be beneficial in coping with the disease while maintaining NLR innate immune responses in CAPS patients who respond to HSP90β inhibition.

In conclusion, this study uncovers the specific role of SGT1-HSP90β in the assembly of the NLRP3 autoinflammasome in humans. This finding emphasizes the possibility of different mechanisms leading to NLRP3 oligomerization. Therefore, further research aimed at identifying these mechanisms and understanding their role in NLRP3 oligomerization may open up avenues for targeted therapeutic approaches in patients with disorders associated with dysregulated NLRP3.

## MATERIALS AND METHODS

### Patient inclusion and human samples

Healthy donor peripheral blood was obtained from Red Cross Switzerland (transfusion interregional CRS) under project number P_247 (table S1). Use and withdrawal of patient peripheral blood were approved by the ethical committee of canton Vaud (CER-VD) with project number 2018_00288 and ethical committee of G. Gaslini Institute (Biobanca per malattie reumatiche e autoinfiammatorie). MG and SV were classified as mild-moderate CAPS (previously FCAS and MWS) or severe CAPS (previously CINCA/NOMID) according to the absence or presence of early-onset hearing loss/central nervous system involvement and skeletal dysplasia.

### Cells and reagents

U937 (male) cells were cultured in RPMI 1640 Glutamax (Gibco) supplemented with 10% fetal calf serum (FCS; Gibco) and 1% penicillin-streptomycin (Gibco) at 37°C and 5% CO_2_. Human embryonic kidney (HEK) 293T (female) cells were cultured in Dulbecco’s modified Eagle’s medium Glutamax (Gibco) supplemented with 10% FCS (Gibco) and 1% penicillin-streptomycin (Gibco) at 37°C and 5% CO_2_. Cells were regularly tested for mycoplasma by polymerase chain reaction (PCR) analysis of culture supernatants (GATC Biotech). PMA (524400), nigericin (N7143), propidium iodide (PI) (P4170), KunB31 (SML2273), GM (SML1278), and Hoechst (B2261) were purchased from Sigma-Aldrich. Cycloheximide (A0879) was purchased from AxonLab. Disuccinimidyl suberate (A39267) was purchased from Thermo Fisher Scientific. Z-vad-fmk (4026865) was purchased from Bachem. Alum (tlrl-aloh) and LPS (tlrl-eklps) were purchased from InvivoGen. Doxycycline (A2951), ampicillin (A0839), and kanamycin (A1493) were purchased from AppliChem. E. cloni (60107) and ENDURA (60240) chemically competent bacteria were purchased from Lucigen.

### Antibodies

Antibodies against pro–IL-1β (12242) and cleaved IL-1β (83186) were purchased from Cell Signaling Technology. Antibodies against tubulin (AG-27B-0005-C100), caspase-1 (AG-20B-0048-C100), and NLRP3 (AG-20B-0014) were purchased from Adipogen. Antibodies against gasdermin-D (ab210070), HSP90β (ab53497), and HSP90α (ab79849) were purchased from Abcam. Anti-SGT1 (612104) was purchased from BD Biosciences. Anti–vesicular stomatitis virus glycoprotein (VSVg) (V4888) was purchased from Sigma-Aldrich. Anti-ASC (sc-22514) was purchased from Santa Cruz Biotechnology. Anti-mouse–horseradish peroxidase (HRP) (115-035-146), anti-rabbit–HRP (111-035-144), and anti-human–HRP (709-035-149) were purchased from the Jackson Laboratory. Anti-rabbit–Alexa Fluor 647 was purchased from Thermo Fisher Scientific.

### Plasmids and molecular biology

See table S2 for primer sequences. NLRP3 WT, NLRP3 R260W, and MEFV M694V DNA were amplified using primers including AttB sites for BP cloning. BP reaction was performed using the PCR product and pDONR-221 to create pEntry plasmids. Point mutations were introduced on pEntry by site-directed mutagenesis. LR reactions were performed using pEntry and pINDUCER-21 to create doxycycline-inducible lentiviral constructs. SGT1a/b (provided by A. Filipek) and HSP90β (Addgene, 177659) plasmids were used as templates to clone SGT1a, SGT1b, or HSP90β into pEF1a-IRES-BFP (provided by M. Thome). sgRNA sequences were annealed, Esp3I-digested (BioLabs), gel-purified (Cytiva kit), and ligated into pLentiCRISPRv2-Puro or pLentiCRISPRv2-Blast using T4 DNA ligase (Thermo Fisher Scientific).

### Genome-wide CRISPR knockout screening

The Toronto Knock-Out library (*41*) and virus preparation were done according to the accompanying online manual (https://addgene.org/pooled-library/moffat-crispr-knockout-tkov3/). Cells (200 × 10^6^ to 300 × 10^6^) were thrice treated with doxycycline for 48 hours. Dead cells were removed from the culture by magnetic cell sorting separation, and genomic DNA was isolated from the surviving cells. As control, genomic DNA was isolated from 20 × 10^6^ cells and was not treated with doxycycline. Subsequent PCR reactions were performed according to the TKOv3 protocol, and the product was sent for deep-sequencing analysis by HiSeq 4000. MaGeCK analysis of sequencing counts resulted in individual sgRNA scores (data S2) as well as gene scores (data S1) for enrichment within treated cells versus control cells. The raw data for the screen presented in this publication have been deposited online (PRJNA896146).

### sgRNA design

Gene-targeted sgRNA sequences were designed using the CRISPRseek package of Bioconductor (version 3.6) on R. See table S2 for sgRNA sequences used in this study.

### Inflammasome activation

Typically, 1 × 10^6^ cells were plated in Opti-MEM (Gibco), primed with 0.1 μM PMA overnight, and subsequently stimulated for inflammasome activation. For gain-of-function PYRIN or NLRP3 inflammasome activation, cells were treated with doxycycline (2 μg/ml) overnight together with PMA priming. For WT NLRP3 inflammasome activation, the following stimuli were used: nigericin (5 μg/ml or indicated, 1 hour) or alum (indicated concentration, 6 hours). KUNB31 (1 μM) or GM (0.2/1 μM) or vehicle control was added 30 min before LPS stimulation and doxycycline (2 μg/ml) treatment.

Cellular supernatants were collected and processed for protein precipitation and/or ELISA. Cells were lysed in Laemmli buffer [50 mM tris-HCl (pH 5.8), 2% SDS, 10% glycerol, 12.5 mM EDTA, and 0.02% bromophenol blue] supplemented with 100 mM dithiothreitol, incubated at 95°C for 5 min, and stored at −20°C until use. For analysis of ASC oligomers, cells were collected and processed for protein cross-linking. For speck analysis, cells were collected and processed for imaging flow cytometry.

### Pulse chase

Four million cells were plated in six-well plates treated with doxycycline (0.5 μg/ml overnight). Cells were treated with cycloheximide (10 μg/ml) at time 0 hour. Cells were collected and lysed in Laemmli buffer [50 mM tris-HCl (pH 5.8), 2% SDS, 10% glycerol, 12.5 mM EDTA, and 0.02% bromophenol blue] following the chase time (from 0 to 24 hours).

### Protein precipitation

One hundred microliters of chloroform and 500 μl of methanol were added to maximum 600 μl of cellular supernatant, vortexed, and centrifuged for 3 min at 13,000*g* and 4°C. The upper phase was removed, leaving the white protein disc intact, after which 500 μl of methanol was added and mixed into the samples by inverting the tubes. After centrifugation for 3 min at 13,000*g* and 4°C, the supernatants were removed, and pellets were air-dried under the chemical hood for 5 min. Protein pellets were lysed in 60 μl of Laemmli buffer, incubated at 95°C for 5 min, and stored at −20°C until use.

### Immunoblotting

Samples were separated by SDS–polyacrylamide gel electrophoresis (SDS-PAGE) and transferred onto nitrocellulose membranes (Millipore) by wet immunoblotting. Membranes were blocked with 5% nonfat milk powder in phosphate-buffered saline (PBS) 0.1% Tween 20 (AppliChem) for 1 hour and subsequently incubated with a primary antibody for 16 hours at 4°C. After washing, membranes were incubated with HRP-coupled secondary antibody for 1 hour at room temperature followed by analysis on chemiluminescence imaging system (Fusion Solo) using enhanced chemiluminescence (ECL; Cytiva).

### Immunoprecipitations

HEK293 cells were transiently transfected with empty vector, NLRP3 WT, or NLRP3 R260W constructs in 12-well plates (Lipofectamine 2000, Invitrogen) and analyzed 24 hours after transfection. For coimmunoprecipitation of transiently transfected HEK293 cells, cells were washed and lysed in lysis buffer NP-40 [0.2% NP-40, 150 mM NaCl, and 20 mM tris (pH7.4)] supplemented with protease inhibitors using PIs 1X, Naf (5%), Na_4_P_2_O_7_ (5%), and Na_3_VO_4_ (1%). Cleared lysates were subjected to immunoprecipitation by incubating with immobilized antibodies anti-Flag coupled beads and Sepharose beads (Sigma-Aldrich, 6B100) as indicated for overnight at 4°C. Following extensive washing with lysis buffer, bound proteins in Laemmli sample buffer were separated by SDS-PAGE, transferred to polyvinylidene fluoride membranes, blocked [5% nonfat dry milk, 0.1 M tris-buffered saline (pH 7.4), and 0.1% Tween 20], and analyzed by immunoblotting as indicated using HRP-conjugated secondary antibodies and ECL detection (Cytiva).

### ASC cross-linking

Samples were processed as previously described (*42*). In short, 5 × 10^6^ to 6 × 10^6^ cells were treated with doxycycline overnight and lysed by shearing through a 21-gauge needle. Lysates are subjected to several centrifugation steps to ultimately pellet the ASC oligomers. The pellets are cross-linked using 4 mM disuccinimidyl suberate at room temperature for 30 min. Samples were then centrifuged, the supernatant was removed, and the cross-linked pellets were resuspended in sample buffer, boiled, and analyzed by immunoblotting.

### Enzyme-linked immunosorbent assay

Levels of human IL-1β were measured in cell supernatants using Affymetrix eBioscience human IL-1β ELISA Ready-Set-Go! (second generation) (reference no. 88-7261-88, lot no. 4290534). ELISA was performed according to the manufacturer’s instructions.

### Flow cytometry

Cells were collected and washed twice in PBS containing 2% FCS (Gibco). Next, cells were exposed to PI (50 μg/ml) and immediately analyzed using a Cytoflex (Beckman Coulter). Analysis was done using FlowJo version 10 software.

### Imaging flow cytometry

Cells (4 × 10^6^) were treated with nigericin (10 μg/ml, 5 hours) or treated with doxycycline overnight to induce NLRP3-R260W in the presence of 5 μM z-vad to block cell death. Cells were collected and washed twice in PBS containing 2% FCS (Gibco). Next, cells were fixed and permeabilized (Fix/Perm kit BD Biosciences) and subsequently stained with anti-ASC antibodies for 30 min, washed twice, stained with anti-rabbit–Alexa Fluor 647 antibodies for 30 min, washed twice, and taken up in PBS (2% FCS) to be used for measurement using Amnis ImageStream^X^ Mark II (Merck Millipore). Hoechst was added for nuclear stain. A minimum of 50,000 cells were measured for each condition. Analysis was done using Amnis IDEAS V6.2 software and FlowJo version 10 software.

### PBMC isolation

PBMCs from patients or healthy donors were separated from peripheral blood by Ficoll Isopaque density gradient centrifugation (GE Healthcare Bio-Sciences AB) and were either used directly or frozen until further experimentation. For inflammasome activation, patient PBMCs were incubated at 37°C and 5% CO_2_ with LPS for 2 hours; for healthy donor PBMCs, nigericin was added for the last 45 min. KUNB31 (1 μM) or vehicle control was added 30 min before LPS stimulation. Supernatants were collected for ELISA. Where indicated, supernatants were collected for protein precipitation, and cells were lysed for Western blot analysis.

### Statistics

Two-sided nonparametric Mann-Whitney *U* tests (comparing two conditions) or nonparametric Kruskal-Wallis tests including Dunn’s correction for multiple comparisons (comparing >2 conditions) were applied using Prism software version 9.1.2 (225). In the case of paired samples, paired *t* tests (comparing two conditions) or two-way analysis of variance (ANOVA) including Bonferroni correction for multiple comparisons (comparing >2 conditions) were applied.
