# Supplementary material for: HSP90β controls NLRP3 autoactivation
Source: Sci Adv. 2024 Feb 28;10(9):eadj6289. doi: 10.1126/sciadv.adj6289 (PMC10901362; doi:10.1126/sciadv.adj6289)
Supplement: Supplementary file 1 — Figs. S1 to S3 Tables S1 and S2 Legends for data S1 and S2 [file sciadv.adj6289_sm.pdf]

Science Advances
AAAS

Supplementary Materials for

HSP90β controls NLRP3 autoactivation

Lotte Spel *et al.*

Corresponding author: Fabio Martinon, fabio.martinon@unil.ch

Sci. Adv. **10**, eadj6289 (2024)
DOI: 10.1126/sciadv.adj6289

The PDF file includes:

Figs. S1 to S3
Tables S1 and S2
Legends for data S1 and S2

Other Supplementary Material for this manuscript includes the following:

Data S1 and S2

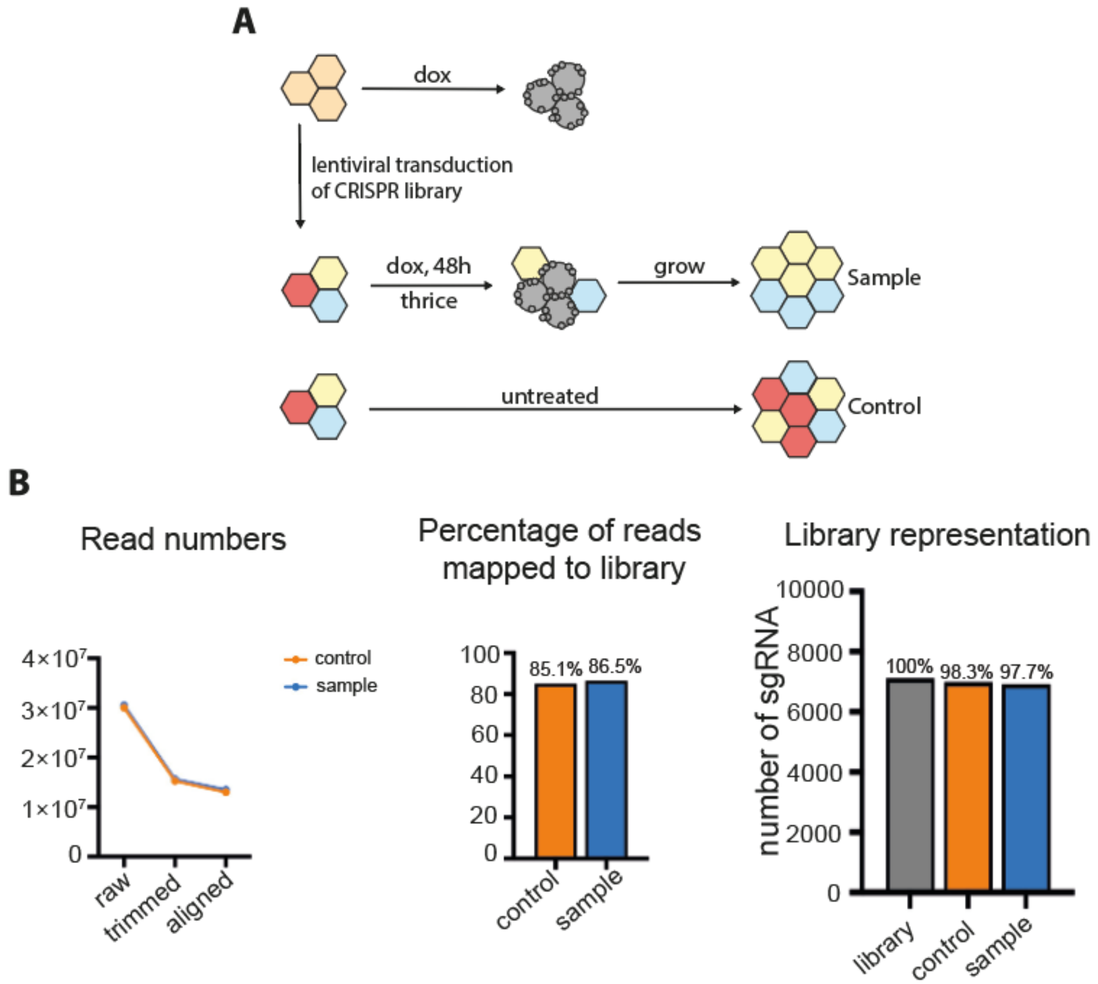

Fig. S1. CAPS survival screen specifications. A) Schematic overview of genome-wide CRISPR knock-out screen performed in U937 NLRP3 R260W cells. A CRISPR library was introduced into the cells by lentiviral transduction generating a mutagenized population. Mutagenized cells were treated with doxycycline and surviving cells were collected (sample) or left untreated (control). Genomic DNA was isolated, sgRNA sequences were amplified by PCR and sequenced. **B)** Specifications of performed CRISPR knock-out screen: number of reads obtained after sequencing for the control and sample; percentage of reads that could be mapped to the sgRNA library; and representation of the total sgRNA library by the control and sample.

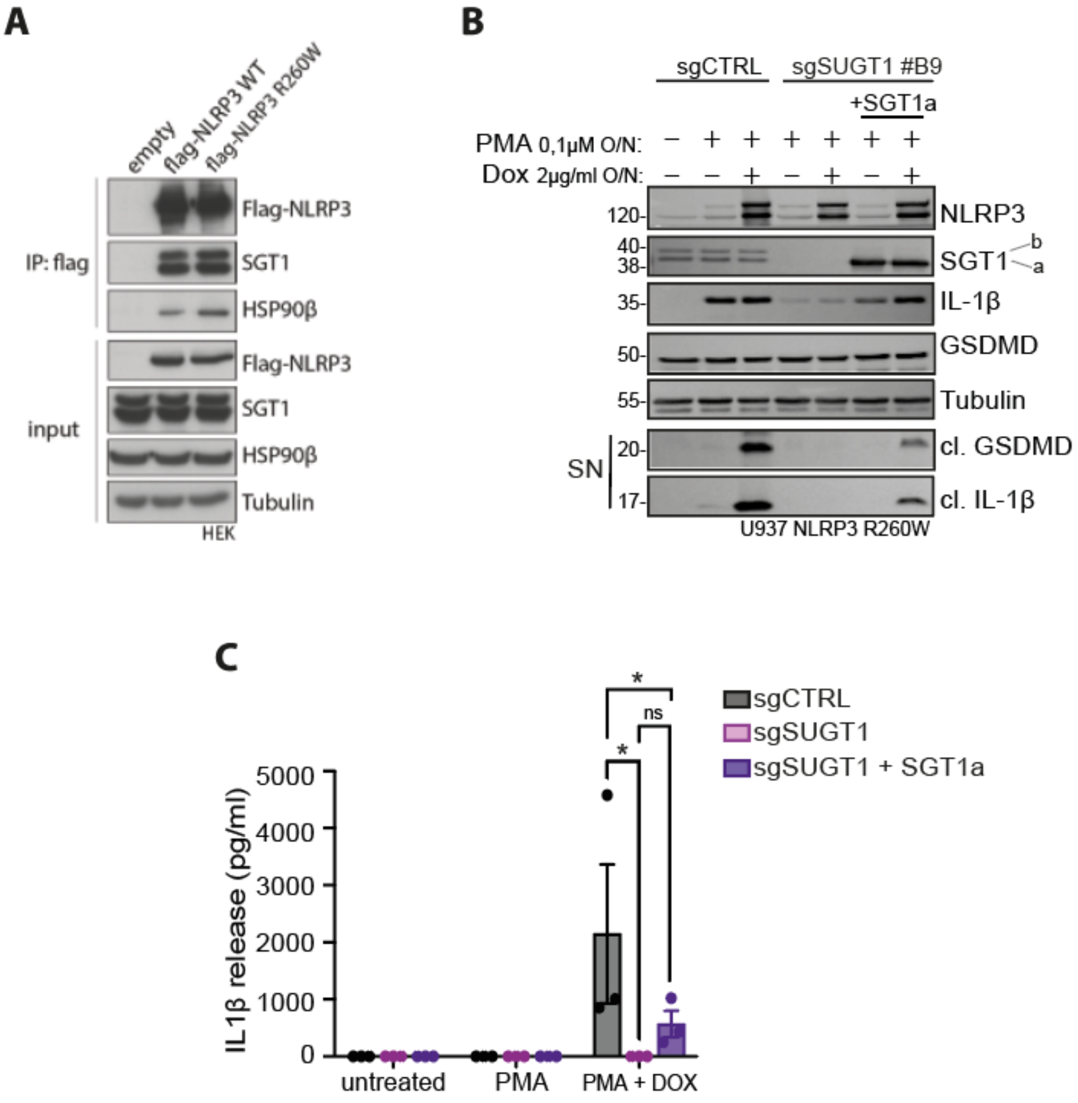

Fig. S2. The SGT1/HSP90β complex is required for NLRP3 inflammasome functionality.

A) HEK-293T cells were transfected with empty plasmid, or plasmids encoding for Flag-NLRP3 or Flag-NLRP3 R260W. Immunoprecipitation with immobilized anti-Flag were analysed by immunoblot for SGT1, HSP90β and Flag-tagged NLRP3 proteins. **B-C)** Inflammasome activation in U937 NLRP3 R260W control cells or depleted for SGT1 (sgSUGT1) or reconstituted with a CRISPR-resistant SGT1a. Cells are treated with PMA and doxycycline overnight. Protein expression and release of inflammasome activation markers are measured by western blot (**B**).
Release of cleaved IL-1β in the supernatant is quantified by ELISA (**C**). Western blots and ELISA are representative of three independent experiments. ELISA data represented as mean ± SD and tested for statistical significance using the non-parametric Mann-Whitney U test ($p \leq 0.05$ is considered significant and indicated with *; ns = not significant).

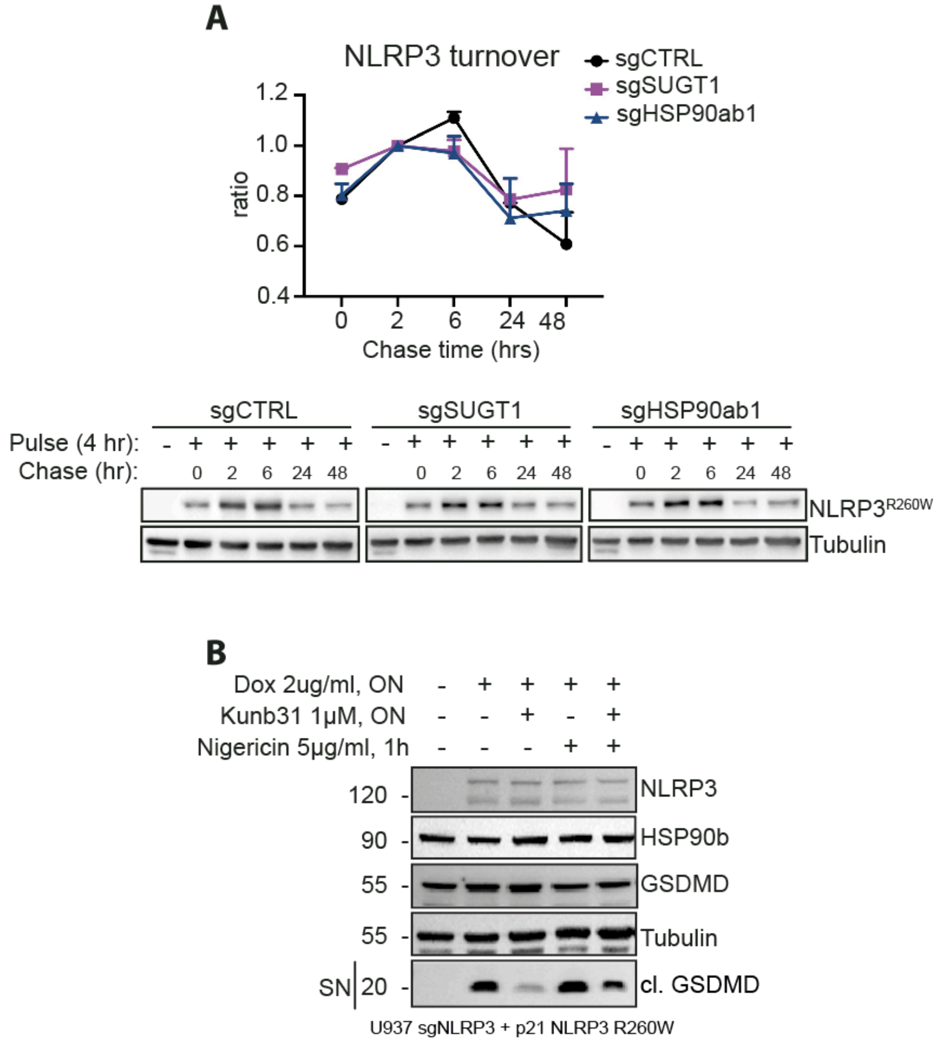

Fig. S3. Impact of HSP90β and SGT1 on NLRP3 protein stability and response to nigericin.

A) NLRP3 turnover in U937 NLRP3 R260W sgCTRL, sgSUGT1 or sgHSP90ab1. Cycloheximide chase is indicated. Representative western-blot for NLRP3 expression from are shown in U937 NLRP3 R260W expressing sgCTRL, sgSUGT1, or sgHSP90ab1. **B)** NLRP3 protein expression and release of inflammasome activation markers are measured by western blot upon treatment with nigericin. Inflammasome activation in U937 deficient in NLRP3 endogenous (sgNLRP3) and reconstituted with a CRISPR-resistant NLRP3 R260W were treated with PMA and dox overnight in the absence or presence of KUNB31 (K31). Then cells were incubated with nigericin for 1 hour. Protein expression and release of inflammasome activation markers are measured by western blot.

Table S1
CAPS patients' samples and mutation associated

Patient	Mutation	Diagnosis
1	R260W	MWS
2	D303N	MWS
3	D303N	MWS
4	D303N	MWS
5	T348M	MWS
6	T348M	MWS
7	T348M	CINCA
8	M406I	CINCA
9	A439V	FCAS
10	N477K	CINCA
11	F523Y	CINCA
12	F523C	FCAS
13	F523C	FCAS
14	E567K	CINCA
15	E567K	CINCA

Table S2 Oligonucleotides used in the study

Oligo name	5' to 3' sequence
NLRP3 BP cloning fwd	GGG GAC AAG TTT GTA CAA AAA AGC AGG CTT CAC CAT GAA GAT GGC AAG CAC CCG CTG CA
NLRP3 BP cloning rev	GGG GAC CAC TTT GTA CAA GAA AGC TGG GTC CTA CCA AGA AGG CTC AAA GAC GAC GGT CA
sgCTRL fwd	CACCGCTTCGAAATGTCCGTTCGGT
sgCTRL rev	AAACACCGAACGGACATTTCGAAGC
sgSUGT1 #B fwd	CAC CGC CTG GGG GTC CTC GTC GAT T
sgSUGT1 #B rev	AAA CAA TCG ACG AGG ACC CCC AGG C
sgHSP90ab1 #A fwd	CAC CGT CTA GGT GAG CCC ATT GGC A
sgHSP90ab1 #A rev	AAA CTG CCA ATG GGC TCA CCT AGA
sgHSP90ab1 #B fwd	CAC CGA CTT TGG TAC CCC TGC CAA T
sgHSP90ab1 #B rev	AAA CAT TGG CAG GGG TAC CAA AGT
sgHSP90aa1 fwd	CTA ATC GAC TTC TTC CAT GCG AGA CG
sgHSP90aa1 rev	CAC CGG CAG TTC TCT AAA AAC ATA A
sgNLRP3 fwd	CAC CGA TCG CAG CGA AGA TCC ACA
sgNLRP3 rev	AAA CTG TGG ATC TTC GCT GCG ATC
SUGT1 resistant to gRNA B fwd	CAC CGC CTG GGG GTC CTC GTC GAT T
SUGT1 resistant to gRNA B rev	AAA CAA TCG ACG AGG ACC CCC AGG C
HSP90ab1 resistant to gRNA A fwd	GTG AGC CCA TTG GCA GAG GTA CCA AAG TGA TCC
HSP90ab1 resistant to gRNA A rev	GGA TCA CTT TGG TAC CTC TGC CAA TGG GCT CAC
INFUSION Cloning SUGT1 into pEF1a-IRES-BFP fwd	GGA ATT TCG ACA TTT CAC CAT GGC GGC TGC TGC AGC AG
INFUSION Cloning SUGT1 into pEF1a-IRES-BFP rev	GGA ATT TCG ACA TTT CAC CAT GGC GGC GGC TGC AGC AG
INFUSION Cloning HSP90ab1 into pEF1a-IRES-BFP fwd	GGA ATT TCG ACA TTT CAC CAT GCC TGA GGA AGT GCA CCA TGG
INFUSION Cloning HSP90ab1 into pEF1a-IRES-BFP rev	AGA TTA ATT AAA TTT GCC TAA TCG ACT TCT TCC ATG CG
NLRP3 point mutation R260W fwd	ACT ATC TGT TCT ATA TCC ACT GTT GGG AGG TGA GCC
NLRP3 point mutation R260W rev	GGC TCA CCT CCC AAC AGT GGA TAT AGA ACA GAT AGT
NLRP3 point mutation L305P fwd	CAA AGG CAC CTT GCG GCT CAT CGA AGC CG
NLRP3 point mutation L305P rev	CGG CTT CGA TGA GCC GCA AGG TGC CTT TG
NLRP3 point mutation A352V fwd	GCA GTT TCT CCA GGA CCA CAG GTC TCG TG
NLRP3 point mutation A352V rev	CAC GAG ACC TGT GGT CCT GGA GAA ACT GC
NLRP3 point mutation L353P fwd	GCT GCA GTT TCT CCG GGG CCA CAG GTC TC
NLRP3 point mutation L353P rev	GAG ACC TGT GGC CCC GGA GAA ACT GCA GC
NLRP3 point mutation F523C fwd	GGC AAA GAA CTC CTG GCA AGT CAT GTG GAT GAA GC
NLRP3 point mutation F523C rev	GCT TCA TCC ACA TGA CTT GCC AGG AGT TCT TTG CC
NLRP3 point mutation E567K fwd	AAA AAT CAA ATA CCC CTT TTT GAA TTT GCC ATA GTT TTC CAG AAG GA
NLRP3 point mutation E567K rev	TCC TTC TGG AAA ACT ATG GCA AAT TCA AAA AGG GGT ATT TGA TTT TT

Data S1. (separate file)
MaGeCk analysis of the screen results including genes rank

Data S2. (separate file)
MaGeCk analysis of the screen results including single guide RNAs rank
